# Differences in the Loin Tenderness of Iberian Pigs Explained through Dissimilarities in Their Transcriptome Expression Profile

**DOI:** 10.3390/ani10091715

**Published:** 2020-09-22

**Authors:** Miguel Ángel Fernández-Barroso, Carmen Caraballo, Luis Silió, Carmen Rodríguez, Yolanda Nuñez, Fernando Sánchez-Esquiliche, Gema Matos, Juan María García-Casco, María Muñoz

**Affiliations:** 1 Centro Nacional de I+D del Cerdo Ibérico (CENIDCI), INIA, 06300 Zafra, Spain; carmen.caraballo@inia.es (C.C.); garcia.juan@inia.es (J.M.G.-C.); 2 Departamento de Mejora Genética Animal, INIA, 28040 Madrid, Spain; luis.siliolopez@gmail.com (L.S.); mrodriguezvaldovinos@gmail.com (C.R.); nunez.yolanda@inia.es (Y.N.); mariamm@inia.es (M.M.); 3 Sánchez Romero Carvajal—Jabugo, SRC, 21290 Huelva, Spain; fernando.sanchez@osborne.es (F.S.-E.); gema.matos@osborne.es (G.M.)

**Keywords:** RNA-seq, transcriptome analysis, Iberian pig, meat tenderness

## Abstract

**Simple Summary:**

The Iberian pig is the most representative autochthonous breed of the Mediterranean region with unique genetic and phenotypic characteristics. The breed has been successfully preserved by its high-quality meat and high-priced products. Tenderness is one of the most relevant meat quality traits, and meat tenderization is influenced by genetic and environmental effects such as pre-slaughter handling and post-mortem conditions. Tenderness could be included in Iberian pig breeding programs, mainly focused on the improvement of premium-cuts percentage, in order to avoid the meat quality decline. A better biological understanding of this trait is needed. In the current study, we analyze the transcriptome of pigs divergent for Warner–Bratzler shear force through RNA-seq technique for the identification, characterization and quantification of candidate genes involved in biological pathways, networks and functions affecting meat tenderness.

**Abstract:**

Tenderness is one of the most important meat quality traits and it can be measured through shear force with the Warner–Bratzler test. In the current study, we use the RNA-seq technique to analyze the transcriptome of *Longissimus dorsi* (LD) muscle in two groups of Iberian pigs (Tough and Tender) divergent for shear force breeding values. We identified 200 annotated differentially expressed genes (DEGs) and 245 newly predicted isoforms. The RNAseq expression results of 10 genes were validated with quantitative PCR (qPCR). Functional analyses showed an enrichment of DE genes in biological processes related to proteolysis (*CTSC*, *RHOD*, *MYH8*, *ACTC1*, *GADD45B*, *CASQ2*, *CHRNA9* and *ANKRD1*), skeletal muscle tissue development (*ANKRD1*, *DMD*, *FOS* and *MSTN*), lipid metabolism (*FABP3* and *PPARGC1A*) and collagen metabolism (*COL14A1*). The upstream analysis revealed a total of 11 transcription regulatory factors that could regulate the expression of some DEGs. Among them, IGF1, VGLL3 and PPARG can be highlighted since they regulate the expression of genes involved in biological pathways that could affect tenderness. The experiment revealed a set of candidate genes and regulatory factors suggestive to search polymorphisms that could be incorporated in a breeding program for improving meat tenderness.

## 1. Introduction

Traditionally, the meat industry and genetic breeding programs have been focused on production traits such as efficient growth rate and carcass leanness. However, an intensive selection for them could alter some porcine muscle characteristics [1] and quality traits [2]. Furthermore, the antagonistic correlation among pigs selected for lean muscle and body growth versus tenderness has been reported [3]. Moreover, muscle from pigs intensively selected for increased lean growth showed lower tenderness [4]. Meat quality plays a key role in determining its commercial value and consumer acceptance, tenderness being one of its most appreciated characteristics.

Meat tenderness is a complex trait influenced by the interaction of many effects, such as genotype, gene expression, environmental conditions, pre-slaughter handling, slaughter and *post-mortem* procedures [5]. Tenderness is moderately heritable, with values ranging from 0.25 to 0.45 both in commercial and autochthonous pig breeds [6,7,8]. In addition, several polymorphisms in candidate genes such as *Calpastatine* (*CAST*) or *Calpain 1* (*CAPN1*) affecting tenderness have been identified [8,9,10]. Its measurement is not easy, the instrumental texture analysis by Warner–Bratzler shear force being one of the most common methods since it is considered an objective and rapid approach [11,12].

One extended approach for identifying candidate genes harboring potential mutations that could partially explain the genetic basis of a particular trait consists in analyzing expression gene changes between individuals divergent for the studied trait. High-throughput RNA sequencing technique (RNA-seq) permits the identification, characterization and quantification of the transcript dataset expressed in any tissue. Previous transcriptome studies using RNA-seq for sequencing the muscle transcriptome of different pig breeds and crossbreds have reported some interesting information about gene expression, biological pathways, networks and functions related with tenderness [13,14,15].

The Iberian pig is the most representative autochthonous breed belonging to the Mediterranean region. This breed is characterized by its high adipogenic potential, voracious appetite, high protein turnover ratio and low lean tissue deposition [16] that are determined by their traditional open-air production system [17] and its unique genetics characteristics [18,19,20]. These features mean that both their fresh meat and derived dry-cured products are vastly appreciated, obtaining a high economic value in Spanish and international markets. The inclusion of different quality traits in the objectives of these programs may be required. The use of molecular genetics techniques is therefore advisable and it has been previously approached in other studies [8,21].

Taking this into account, in the current study we analyze the transcriptome of *Longissimus dorsi* (LD) muscle in Iberian pigs divergent for shear force to identify differentially expressed genes (DEGs) and understand how they are involved in the regulation of biological processes. Therefore, the aims of this study were: (a) to identify and quantify the DEGs and recognize biological processes, pathways, networks and functions in which these genes are involved, (b) to determine transcription regulatory factors influencing the observed gene expression profile, and (c) to propose a set of candidate genes with detected mutations affecting meat tenderness.

## 2. Materials and Methods

### 2.1. Animal Material and Phenotypic Data

The animals used in the current study were commercial castrated male pigs that belonged to an Iberian purebred line closed for approximately 15 years described in a previous study [8]. Animals were fattened in an open-air free-range system (*Montanera*) based on ad libitum intake of acorns and grass. They were managed during three successive years (from 2015 to 2017), being slaughtered at an approximate age of 17 months and with an average slaughter weight of 165 kg. Animal handling was carried out according to the regulations of the Spanish Policy for Animal Protection RD 53/2013, which meets the European Union Directive 2010/63/EU about the protection of animals used in research. Protocols were assessed and approved by the INIA Committee of Ethics in Animal Research, which is the named Institutional Animal Care and Use Committee (IACUC) for the INIA.

*Longissimus dorsi* samples from 892 animals were removed from the carcass after slaughter. A central muscle section of approximately 300 g were separated of each loin for meat determination. These samples were vacuum-packed in nylon/polyethylene bags and stored at −20 °C until analysis. After that, samples were thawed and subsequently cooked by immersion at 70 °C during 1 h in a water bath (VWR, Pennsylvania, USA) [22]. Texture was determined in cooked meat portions following [11] and measured as cooked meat shear force (SFF) by the Warner–Bratzler test (Stable Microsystems TA.XT Plus, Godalming, UK). Eight pieces of 3 cm × 1 cm × 1 cm (length, width and thickness) were cut perpendicular to the muscle fiber direction with a Warner–Bratzler blade (HDP/BSW) and the eight repeated measures were averaged. SFF was measured as kg/cm^2^. The mean of the SFF was 4.33 (SD = 1.10).

The following mixed model was used to estimate breeding values (EBVs) for SFF:

y = Xb + Za + Wsm + e(1)

where **y** is the vector of SFF values corresponding to each animal, **b** represents the vectors of systematic effects in which the slaughter weight was fitted as a covariate, **a** is the vector of the additive genetic effects (EBVs) distributed as *N* (0, ***Aσ^2^_u_***), where ***A*** is the numerator of kinship matrix that allows for the adjustment of the data taking into account the pedigree information, **sm** is the vector of the combined fattening-slaughter batch environmental random effects (19 levels) and **e** the vector including the residual effects. **X, Z** and **W** are the incidence matrices. EBVs were estimated using the TM program [23].

A total of 13 pigs with the most extreme EBVs for SFF, avoiding full and half siblings, were selected. The Tough group contained six individuals showing the highest EBVs, and the Tender group contained the seven ones with the lowest values. The EBV averages were 2.11 (SD = 0.29) and −1.03 (SD = 0.05) for the Tough and the Tender group, respectively, and the corresponding phenotypic mean values for shear force were 9.17 kg/cm^2^ (SD = 1.28) and 2.83 kg/cm^2^ (SD = 0.48).

### 2.2. Transcriptomic Analyses

#### 2.2.1. RNA Extraction, Library Preparation and Sequencing

The individual samples of *Longissimus dorsi* collected after slaughter were introduced on cryogenic tubes, frozen in liquid nitrogen and stored at −80 °C until analysis. RiboPure TM of High-Quality total RNA kit (Ambion, Austin, TX, USA) was used to extract the total RNA, following the manufacturer’s recommendations. RNA was quantified using a NanoDrop equipment (NanoDrop Technologies, Wilmington, DE, USA) and the Agilent 2100 Bioanalyzer device (Agilent Technologies, Santa Clara, CA, USA) was employed to evaluate the RNA integrity (RNA Integrity Number = RIN), the RIN values obtained for all the samples were in the range from 7 to 8.

Paired-end libraries were built using TruSeq SBS Kit v3 (Illumina, San Diego, CA, USA) for each sample. Multiplex sequencing of the libraries was carried out on a HiSeq2000 sequence analyzer (Illumina, Inc, San Diego, CA, USA) with four samples per lane at Centro Nacional de Análisis Genómico (CNAG-CRG; Barcelona, Spain), according to the manufacturer’s instructions. Pair-end reads of 74 bp were generated. The raw sequence data of 12 of the 13 individuals have been deposited in the Gene Expression Omnibus (GEO) database with the accession number: GSE155915. The sample named Tender_ 7 in the present study was already sequenced in a previous study [24] and its sequence data was already deposited in GEO with the accession number GSE116951 and its identification corresponds to the individual H5.

#### 2.2.2. Bioinformatics Analyses

##### Mapping, Assembly and Identification of Novel Isoforms

The quality of raw sequencing data was evaluated with FastQC (Babraham Bioinformatics, http://www.bioinformatics.babraham.ac.uk/projects/fastqc/). The quality parameters measured with this tool corresponds to sequence-read lengths and base-coverage, nucleotide contributions and base ambiguities, quality scores and over-represented sequences. All the samples passed the quality control (QC) parameters: same length, 100% coverage in all bases, 25% of A, T, G and C nucleotide contributions, 50% GC on base content and less than 0.1% of overrepresented sequences. TrimGalore was used to trim the raw sequences through removing the sequencing adaptor and poly A and T tails, setting default values (stringency of 6 bp) and keeping paired-end reads when both pairs were longer than 40 bp. TopHat v2.1.0 [25] was used to map the filtered reads against the pig reference genome (Sscrofa11.1). Cufflinks v2.2.1 [26] was employed to assemble and quantified the transcripts in fragments per kilobase of transcript per million (FPKM) mapped reads. The normalized expression data have been deposited in the GEO database with the accession number GSE155915 and GSE116951. Cuffcompare tool (from Cufflinks) was used to identify isoforms not described so far. It was run using Ensembl (Sscrofa11.1) transcriptome annotation as a reference to assess the accuracy of the predicted Cufflinks mRNAs or gene models. Finally, a list with all class codes of the transcript was reported by Cuffcompare.

##### Differential Expression Analyses

Cuffdiff was used to quantify the expression values and carry out the differential expression analyses between the Tough and Tender groups of annotated genes and novel described isoforms. Cuffdiff was run setting the bias correction (-b option) and the rescue method for multireads (-u option). The remaining parameters were established as default. These genes and novels isoforms were filtered according to the following criteria: an average group expression greater than 0.5 FPKM and a fold change value (FC) of the expression differences between the Tender and Tough groups’ ≤ 0.67 and ≥ 1.5. Besides, R package *q*-value [27] was used to correct multiplicity of test, where *q*-value provides a method to control the false discovery rate (FDR), which is the proportion of false positives among all positive results, and genes and new isoforms were considered as differentially expressed with a *p*-value ≤ 0.05 and *q*-value *≤* 0.10.

##### Gene Functional Classification, Network and Pathway Analyses

Gene Ontology (GO) information was used to analyze the functionality of the DEGs between the Tough and Tender groups. The biological interpretation of the data was carried out using FatiGO browser from Babelomics 5 (Babelomics 5, http://babelomics.bioinfo.cipf.es/). The potential interactions between the proteins codified by the DEGs and clustering through the Markov Cluster Algorithm (MCL) were studied using STRING tools v11.0 [28].

Ingenuity Pathway Analysis (IPA, Ingenuity Systems, Qiagen, CA, USA) bioinformatics tool was used to identify and characterize biological functions, gene networks, canonical pathways and transcription regulatory factors affected by the DEGs. This software assesses the significant association between the data set of DEGs and canonical pathways. In addition, it builds networks with the set of genes using the records harbored in the Ingenuity Pathways Knowledge Base. Potential regulators of differential gene expression were also identified using the tools “upstream regulators” and “causal networks”; these tools analyze if the potential transcriptional factors and upstream regulators contained in the Ingenuity Knowledge Base repository activate or inhibit the differential gene expression pattern through the estimation of a z-score. This z-score statistically measures the significance between the regulator and its potential targets and the direction among them [29].

### 2.3. RNA-seq Results Validation by Quantitative PCR

RNA from the 13 animals used in RNA-seq study was used to perform the technical validation of the RNA-seq experiment through measuring the expression of 10 genes (*MSTN*, *ANKRD1*, *ACTC1*, *MX1*, *FOS*, *COL1A1*, *ELOVL6*, *SSH2*, *NOS2*, *and IRF1*) with quantitative PCR (qPCR). Six genes were selected from de list of DEGs (upregulated in the Tough group or in the Tender group) and four were not differentially expressed between the Tough and Tender (two of them showed low expression and the other two showed a medium-high expression). In a first step, first-strand cDNA synthesis was carried out using Superscript II (Invitrogen, Life Technologies, Paisley, UK) and random hexamers in a total volume of 20 μL using 1 μg of total RNA, according to the manufacturer’s instructions.

Primer pairs used for quantification were designed using Primer-Blast (NCBI, https://www.ncbi.nlm.nih.gov/tools/primer-blast/) from the available GENBANK and/or Ensembl sequences, covering different exons in order to assure the amplification of the cDNA. Table S1 shows primer sequences and amplicon lengths. Then, a standard PCR on cDNA for each primer was carried out to verify amplicon sizes. Next, following standard procedures, SYBR Green Mix (Roche, Basel, Switzerland) in a LightCycler480 (Roche, Basel, Switzerland) was used for the quantification, and data analysis was carried out with LightCycler480 SW1.5 software (Roche, Basel, Switzerland). Three technical replicates were run per each sample and dissociation curves were obtained to confirm the specific amplification of each gene. A total of four cDNA dilutions were carried out in order to build a standard curve and estimate PCR efficiency. Mean crossing point values (Cp) were used for performing the statistical analyses. The Cp value is the PCR cycle number at which the sample’s reaction curve intersects the threshold line. Genorm software was used to calculate the stability of the endogenous genes *ACTB* and *B2M* [30] and these endogenous genes were used to normalize the data through normalization factors. Relative quantities of DEGs were divided by normalization factors, which were the geometric means of the two reference gene quantities. Finally, the technical validation was performed studying the Pearson correlation between the expression values obtained from RNA-Seq data (FPKM) and the normalized gene expression data obtained by qPCR and calculating the concordance correlation coefficient (CCC) [31] between fold change values estimated from RNA-Seq and qPCR expression measures by the two techniques for the 10 genes.

## 3. Results

### 3.1. Characterization of Longissimus Dorsi Transcriptome

In the present study, the *Longissimus dorsi* tissue transcriptome of 13 animals was characterized with the RNA-seq technique. All samples passed the quality control. We obtained 1474 million raw paired-end reads and 1457 million reads after trimming and filtering. A range from 92.80% to 94.50% of the reads mapped to the porcine reference genome was used (Table S2). These results agree with the previous study carried out in the same tissue of pigs from the same population but in different individuals (except L7) [24] and with a study carried out in *Semimembranosus* muscle tissue on Large White pigs [32].

Table S3 shows the classification of the transcripts in relation to the Ensembl annotated porcine genes and a total of 109,085 transcripts expressed in the 13 animals were detected by Cufflinks tool. The potentially new isoforms annotated represents a 43.29% of the transcripts. The intergenic transcripts predicted were a 7.14% of the total and the percentage of transcripts falling entirely within a reference intron was a 14.88%, this could be related with intron retention events, incorrect annotation of exons, errors or missing prediction of isoforms [33].

Expression distribution values of the 25,878 genes annotated in the pig genome reported with Cuffdiff are shown in Figure S1, where the distribution of gene expression levels in FPKMs was similar for the Tough and Tender groups.

### 3.2. Differential Expression Analyses

The differential expression analyses revealed a total of 200 annotated genes and 245 newly predicted isoforms differentially expressed between the Tough and Tender groups. A total of 118 annotated genes were upregulated in the Tender group (FC ≤ 0.67) while 82 genes were upregulated in the Tough group (FC ≥ 1.5) (Table S4). Besides, 128 newly predicted isoforms presented higher expression in the Tender group and 117 in the Tough group. Regarding the fold change, values ranged from 0.04 to 8.83. The genes with the highest expression differences between groups were *GBP1 (FC =* 0.09, *p*-value = 5 × 10^−5^*,* overexpressed in the Tender group) and *FAM180B* (FC = 8.83, *p*-value *=* 3.5 × 10^−4^, overexpressed in the Tough group) (Table 1). The further functional analyses were focused on differentially expressed annotated genes.

### 3.3. Gene Functional Analysis

The GO enrichment analyses carried out with FatiGO identified 457 GO biological processes (GO_BP_) and two GO_SLIM_ (cut-down versions of the GO ontologies containing a subset of the terms in GO) enriched in DEGs (Table S5). Table 2 shows a summary of significant overrepresented pathways that could be more related with meat tenderness. For instance, these pathways are involved in skeletal muscle tissue development (GO: 0007519, 10 genes), regulation of muscle system process (GO: 0090257, seven genes), collagen metabolic process (GO: 0032963, six genes), regulation of calcium ion transport (GO: 0051924: six genes), c-Jun N-terminal kinase (JNK) cascade (GO: 0007254, six genes), actin-myosin filament sliding (GO: 0033275, four genes), skeletal muscle tissue growth (GO: 0048630, two genes), positive regulation of proteolysis involved in cellular protein catabolic process (GO: 1903052, four genes) and actomyosin structure organization (GO: 0031032, three genes).

Subsequently, STRING tools v11.0 revealed networks of protein–protein interactions codified by annotated DEGs and novel predicted isoforms (Figure S2). Five differentiated clusters were observed, two clusters comprised proteins codified by DEGs overexpressed in the Tender group (clusters 1 and 2) and three clusters comprised proteins codified by DEGs upregulated in the Tough group (clusters 3, 4 and 5). Cluster 1 constituted TAP1, PSMB8, PSMB9, TNFRSF12A and SPOPL associated with the cellular amino acid metabolic process and protein and peptide regulations. Cluster 2 constituted CXCL9, CXCL10, ADRA2C, CCL4, CTSC, FCN2, C2, C3, C4 and C1QA associated with skeletal muscle tissue development, calcium ion transport and proteolysis regulation. Cluster 3 constituted COL1A1, COL1A2, COL12A1 and COL14A1 associated with cellular and collagen metabolic processes. Cluster 4 constituted MYLK2, MYLK4 and PAK1 associated with skeletal muscle tissue development and protein autophosphorylation. Cluster 5 constituted MSTN, DMD and AQP4 associated with skeletal muscle tissue development.

**Figure 1:**
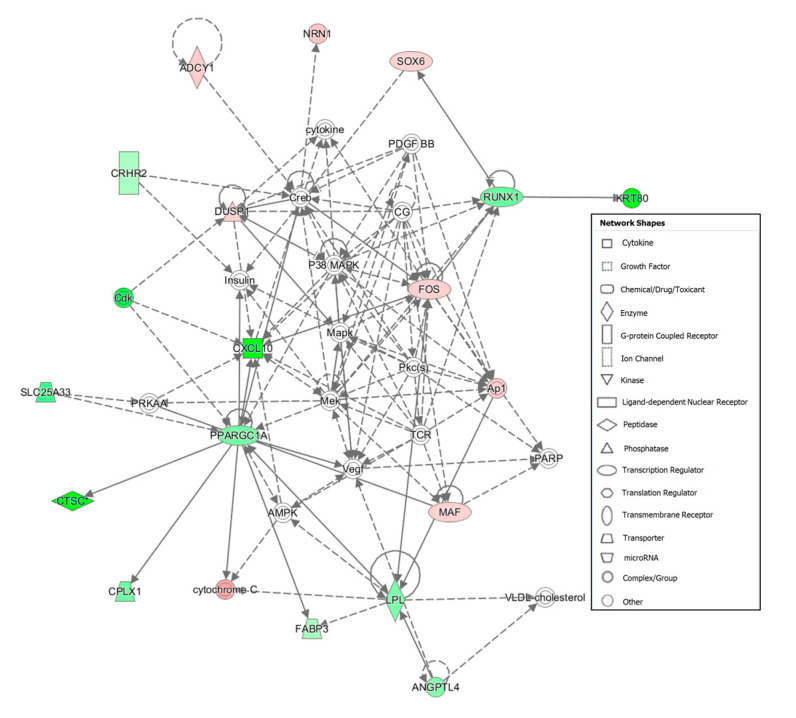
Gene network #5: Connective Tissue Development and Function, Lipid Metabolism, Tissue Morphology. Genes up-regulated and down-regulated in the Tender group are represented in green and red colors, respectively.

**Figure 2:**
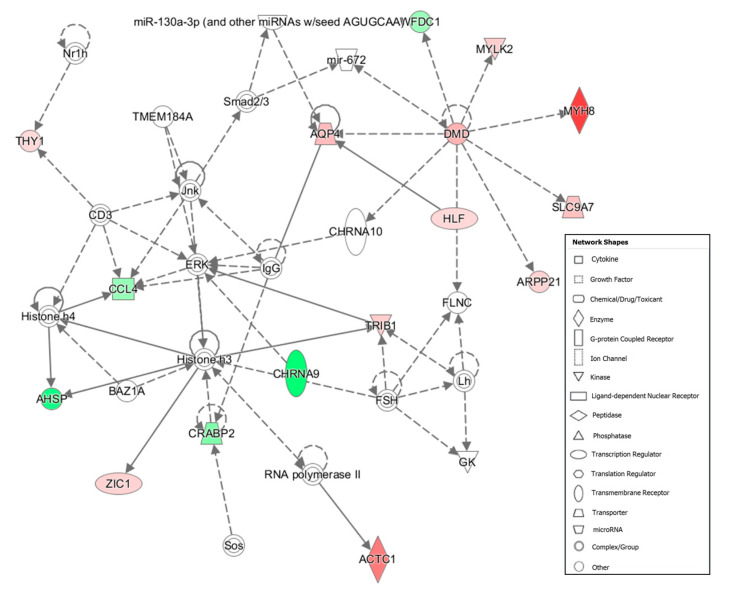
Gene network #6: Cell Morphology, Cellular Assembly and Organization, Cellular Function and Maintenance. Genes up-regulated and down-regulated in the Tender group are represented in green and red colors, respectively.

Furthermore, functional analysis carried out with IPA software revealed 12 networks enriched in DEGs (Table S6). In these analyses, the networks were ranked according to their size and the number of targeted genes and a network score was assigned. This score is estimated as the negative logarithm of the *p*-value calculated by Fisher’s exact test. The two networks closely related with tenderness and muscle development are showed in Table 3. Functions described in gene network #5 (Figure 1) are related with *connective tissue development* *and function, tissue morphology and lipid metabolism* and in gene network #6 (Figure 2) with *cell morphology, cellular assembly, organization, function and maintenance.* Other networks which enclosed relevant DEGS among the Tender and Tough groups are represented in Figure S3 (network #8) and Figure S4 (network #9).

#### 3.3.1. Canonical Pathways Analysis

An additional functional interpretation of global gene expression differences was carried out. A total of 86 canonical pathways were significantly enriched (*p*-value < 0.05) in the dataset of 200 DEGs (Table S7). Furthermore, the analysis reported 12 pathways with assigned z-score, predicting an overall increase in the activity of the pathway in the Tough group when z-score was greater than zero and an overall increase of the pathway in the Tender group when z-score was less than zero, but none of them were significantly activated or inhibited (z-score > 2 or < −2, Table 4). For instance, RhoA Signaling, PPARα/RXRα Activation and White Adipose Tissue Browning showed a trend for activation in the Tender group. On the other hand, some of the pathways presented a positive z-score indicating a trend for activation in the Tough group, as Actin Cytoskeleton Signaling, ILK signaling, Tec Kinase Signaling, Integrin Signaling and Rho Family GTPases. With regards to other significantly enriched canonical pathways (*p*-value < 0.05, Table S7), it is worth mentioning calcium signaling (*p*-value = 0.02) and IGF-1 signaling (*p*-value = 8.7 × 10^−3^).

We found some evidence that canonical pathways related to cell cycle, motility, organization and function, apoptosis, immunological system and lipid metabolism were enriched in the Tender group and that pathways related to skeletal muscle development and growth, such as with cell function, movement and survival presented a trend for activation in the Tough group.

#### 3.3.2. Transcription Regulatory Factors

The upstream analysis and regulator effect tools of IPA were applied to analyze potential transcription regulatory factors of DEGs involved in different molecular processes, which may explain the differential expression observed between the Tender and Tough group.

**Figure 3:**
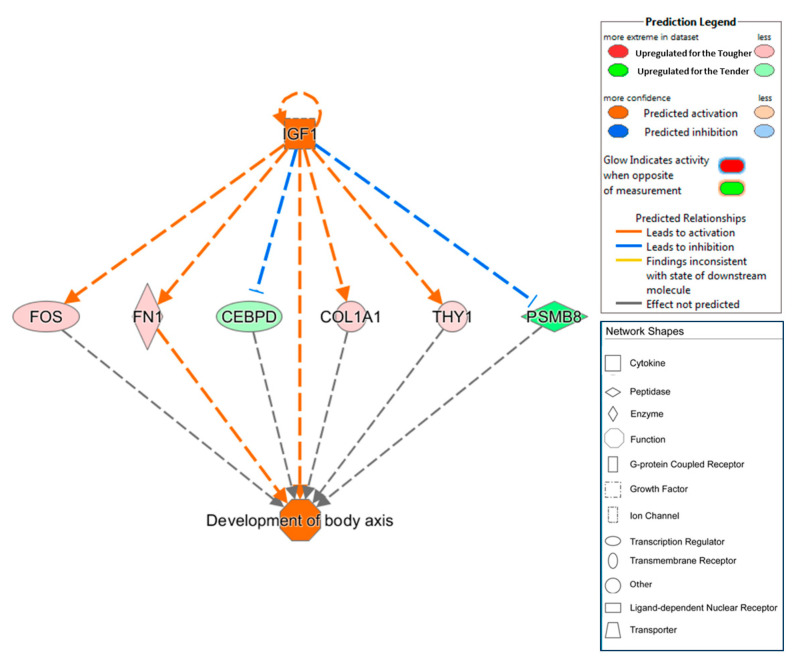
Regulator effects network predicted as activated in purebred Iberian pigs fattened in a free-range system. In the upper tier is *IGF1* (predicted to be activated, orange color). In the middle, there are the genes whose expression changes in response to the activation of *IGF1* (green upregulated for the Tender group and red upregulated for the Tough group). Dashed lines between *IGF1* and DEGs represent the interactions, predicted to be activated (orange lines) or predicted to be inhibited (blue lines). In the lower tier, the expected phenotypic activate function (development of body axis, orange color) is shown.

A total of 860 transcriptional regulators were identified (*p*-value < 0.05, Table S8). Moreover, the sense of activation state was statistically significant predicted for 11 of them (z-score > 2 or z-score < −2, Table 5), five were activated in the Tender group (z-score < −2, KLF11, IL4, PPARG, OGT and NOS2) and six were activated in the Tough group (z-score >2, IGF1, VGLL3, SEMA7A, PTH, TRIM24 and SATB1). Regulator effect tool predicted just one regulator effect network (Figure 3). This network represented a causal hypothesis to interpret the regulatory potential mechanism of the upstream regulator (IGF1) in the expression of some DEGs.

### 3.4. RNA-Seq Validation by qPCR

The relative expression of 10 genes was quantified with qPCR in the 13 samples in order to validate the results observed in the RNA-seq technique. We calculated Pearson correlation between RNA-seq and qPCR expression values, their corresponding *p*-values and the CCC. Table 6 shows the results of technical validation, where seven of the total of genes presented a correlation coefficient > 0.7, nine genes showed a significant *p*-value (*p*-value < 0.05) and only *MSTN* gene presented a suggestive significance value (*p*-value = 0.06). The CCC was equal to 0.828, suggesting a substantial general concordance between RNA-seq and qPCR expression values [31]. In addition, the *IRF1* gene showed the highest agreement between methods and *MSTN* gene presented the lowest concordance.

## 4. Discussion

In the present study, functional analysis of DEGs revealed a set of biological processes, canonical pathways and networks, potentially related with tenderness. In the functional enrichment analyses using FatiGO, there were an overrepresentation of processes related with *Proteolysis* such as Positive regulation of proteolysis involved in cellular protein catabolic process (GO:1903052), Cytosolic calcium ion transport (GO:0060401), Regulation of calcium ion transport (GO:0051924) or Actin-myosin filament sliding (GO:0033275); *Skeletal muscle tissue development and growth*: Skeletal muscle tissue development GO:0007519), Muscle cell development (GO:0055001), Skeletal muscle cell differentiation (GO:0035914), or Regulation of muscle system process (GO:0090257); *Lipid metabolism*: Lipid homeostasis (GO:0055088), Lipid storage (GO:0019915) or Positive regulation of lipid storage (GO:0010884) and *Collagen metabolic process*: Collagen metabolic process (GO:0032963), Collagen fibril organization (GO:0030199) or Collagen biosynthetic process (GO:0032964) (Table 2 and Table S5).

Next, the most relevant DEGs and their potential implications in the aforementioned biological pathways and processes will be detailed.

### 4.1. Proteolysis Process

It is well-known that the proteolytic system has a key role in meat tenderization [34], which is related with the degree of post-mortem alteration of proteins and muscle structure [35]. Several proteases such as calpains, calpastatins, cathepsins, caspases and kinases are involved in the meat tenderization process [34]. During the conversion of muscle to meat, cathepsins degrade actomyosin binding [34] and the weakening of the strong actomyosin interaction imply the widening of sarcomeres. Then, calpains are more able to hydrolyze associated proteins, allowing proteolysis and influencing the maturation of muscle [36]. In our study, a higher expression of the *Cathepsine C* gene (*CTSC*) in the Tender group was observed (Table 1). The functional analyses revealed that this gene is associated with the positive regulation of proteolysis (GO:1903052, Table 2). In a study comparing muscle expression in the Casertana pig breed with two commercial breeds, an overexpression of *CTSC* in Casertana muscle was also observed [37]. Like the Iberian breed, Casertana is an autochthonous breed characterized for having better meat quality than commercial ones. These results support that higher expression of *CTSC* is associated with a higher activation of the proteolysis process favoring the meat tenderization. Besides, in a variant calling analyses based on RNA-seq data of two Polish pig breeds divergent for meat tenderness, variants with different genotype distribution between breeds on *CTSC* gene were detected [38]; however, any association analyses between the genetic variants identified and tenderness have been carried out so far.

Furthermore, *Ras Homolog Family Member D (RHOD)* is overexpressed in the Tender group and codifies for a protein involved in reorganization of the actin cytoskeleton. Our functional analysis showed that *RHOD* was involved on actin filament organization GO_BP_ (GO: 0061572) (Table 2). *RHOD* gene maps on the porcine chromosome 2 (5.39 Mb) within a quantitative trait loci (QTL) for shear force detected in the F2 of a Duroc x Pietrain crossbred [39]. In vitro studies have revealed that the interference of RHOD protein produces a higher cell attachment and diminishes cell migration [40]. Therefore, higher expression of the *RHOD* gene could ease the degradation of actin cytoskeleton during proteolysis.

Two of the most overexpressed genes in the Tough group were *Myosin Heavy Chain 8 (MYH8)* and *Actin Alpha 1, Skeletal Muscle (ACTC1)*, both enclosed in functional network #6 (Table 3), and functional analysis revealed that *MYH8* and *ACTC1* play a relevant role in GO_BP_ as actin-myosin filament sliding, structure organization and contraction (Table 2) and *ACTC1* is also involved in muscle cell development (GO: 0055001). MYH8 protein is related with functions as skeletal muscle contraction, ATPase activity ([41] and actin filament binding [42]. A higher expression of this gene was related with muscle hypertrophy in a transcriptome analysis on Canadian double-muscled Large White pigs, which are characterized by having a notable muscle mass [43]. *ACTC1* encodes for a protein involved in skeletal muscle development [44] and contributes to the structural integrity of cytoskeleton [45]. Expression differences of *ACTC1* associated to tenderness have been uneven. In a study comparing the transcriptome of *Longissimus dorsi* between Shaziling pig, an autochthonous Chinese pig breed with a high-quality meat than Yorkshire, an overexpression of *ACTC1* was observed in Shaziling pig [46]. However, the study was carried out in 25-day-old pigs and the results could be different in older animals. On the other hand, in a study comparing the *Longissimus dorsi* transcriptome of male and female Qinchuan cattle individuals, in which females have tenderer meats, a down-regulation of *ACTC1* gene was observed. In our study, the overexpression of *ACTC1* is apparently associated with tougher meat.

*GADD45B* gene was overexpressed in the Tough group. This gene encodes for Growth Arrest and DNA Damage Inducible Beta protein, which plays a crucial role in cellular growth arrest and apoptosis, associated with stress signals [47]. The authors of [48] observed a higher expression of *GADD45B* in cattle *Longissimus thoracis* muscle with high ultimate pH values. Alteration of pH implies changes in the regulation of calcium transport pathways into the cellular sarcoplasm. When pH muscle is at isoelectric point (5.2 to 5.5) an increase in calcium concentration in the cell is produced, causing a rise of calpain activity [49,50], which degrades myofibrillar and cytoskeletal proteins, promoting meat tenderization [51]. A disparity of results regarding the relationship between pH and tenderness has been reported by other authors. While [6] did not observe a phenotypic relation between these traits, [52] determined that the relationship between pH and tenderness depends on the breed. In this study, we do not have pH values and we cannot conclude that the differential expression of *GADD45B* gene among groups could be explained by the pH.

*ACTC1* and *RHOD* codify for proteins involved on ILK and Integrin signaling pathways (Table 4). ILK Signaling is related with cell survival and apoptosis [53] and Integrin Signaling is linked with cell apoptosis and regulation of actin cytoskeleton [54]. Interestingly, [15] reported pathways involved in cellular apoptosis (survival) and stress response as important factors of tenderization. Moreover, apoptosis is considered one of the first steps in development of meat tenderization, inducing biochemical and structural muscle changes [55]. In the same direction that *GADD45B,* *ACTC1* and *RHOD* have been associated with cellular apoptosis.

Other important DEGs that could be involved in proteolysis, with higher expression in the Tender group, were *Calsequestrin 2* (*CASQ2*), *Cholinergic Receptor Nicotinic Alpha 9 Subunit* (*CHRNA9)* and *Ankyrin Repeat Domain 1 protein* (*ANKRD1*). *CASQ2* codifies for a protein involved in calcium store in the sarcoplasmic reticulum and also modulate calcium homeostasis, calcium release and muscle contraction [56]. In the functional analyses, there was an enrichment of the *CASQ2* gene in GO annotations related with calcium transport and muscle contraction (Table 2). Differential expression of *CASQ2* was also observed in several studies contrasting the transcriptome of breeds divergent for several meat quality parameters including tenderness in some cases. These studies compared the muscle transcriptome of Basque vs. Large White [13], Iberian vs. Duroc × Iberian crossbred [57] and Wannanhua vs. Yorkshire breeds [58]. However, in these studies, the highest expression level of *CASQ2* was observed in the breed with the tougher meats. This disagreement could be due to the fact that the expression differences observed in these studies are between breeds divergent for different quality traits and that, in our study, we analyzed the expression differences between Iberian pigs divergent for meat tenderness.

*ANKRD1* gene was proposed as candidate gene for meat quality by [59] since they observed in their study that it could be a transcriptional regulator of myogenesis and of myofibril assembly in porcine LD muscle of Duroc x Pietrain. In the present study, an enrichment of this gene was observed in GO_BP_ related with cellular assembly involved in morphogenesis and myofibril assembly, actomyosin structure organization and sarcomere organization (Table S5). In addition, *ANKRD1* is involved in biological processes related with muscular growth as skeletal muscle tissue development and muscle cell differentiation linked to myogenesis (Table 2). The authors of [13] also observed a higher expression of *ANKRD1* in Large White than in Basque pigs and proposed that ANKRD1 interacts with CASQ2 protein, which regulates calcium homeostasis in skeletal muscle as it was observed in cardiac muscle [56]. The overexpression of both genes in tenderer meat group observed in our study would support this hypothesis.

*Cholinergic Receptor Nicotinic Alpha 9 Subuni*t (*CHRNA9*) was enriched in a biological process related to the regulation of cytosolic calcium concentration (Table S5), and the canonical pathway analysis interpreted that *CHRNA9* is involved in Calcium signaling pathway, together with *ACTC1*, *CASQ2* and *MYH8* genes (Table S7). High expression of *CHRNA9* was associated with tenderer meats in F2 animals from Duroc × Pietrain cross [60]. One more time, a regulation of the calcium releasing to the cytoplasm would have related with proteolytic enzymatic activity and have an influence on meat tenderness.

### 4.2. Skeletal Muscle Tissue Development and Growth

As we mentioned above, the genetic selection of most common European breeds has usually been focused on improving the efficiency of lean tissue growth. The increase in growth rate and lean meat percentage could alter other meat characteristics such as myofiber composition [61] which would have an impact on meat tenderness. Nevertheless, it should be noted that Iberian pigs have not been previously selected for this or other related traits.

Our transcriptome analysis revealed some pivotal DEGs related with cellular and muscle development such as *MSTN*, *DMD*, and *FOS*, were overexpressed in the Tough group. *Myostatin* (*MSTN)* encodes for a protein that inhibits myogenesis. This process consists of the growth and differentiation of muscle. The inhibition or loss of function of this gene produces an increase in muscle and reduced fat mass that have been reported in several animal species as cattle [62] or sheep [63]. In pigs, *MSTN* null mutations generated in Meishan individuals reproduced the double muscle phenotype and meat from pigs homozygous for the mutation was tenderer than the wild-type ones [64]. This study agrees with our results, and both seem to be contradictory since we would expect that animals with higher muscle mass have tougher meat. However, the role paper of MSTN on adipogenesis has to be considered too. Despite this general lower fat mass content, an inhibition of adipogenesis in intramuscular preadipocytes isolated from porcine *Longissimus dorsi* muscles has been observed [65]. In the current study, the intramuscular fat content in the animals with tougher meat was lower (%IMF = 3.38 ± 0.73) than that measured in the group with tenderer meat (%IMF = 7.81 ± 2.29). Therefore, this higher expression in the Tough group could inhibit the adipogenesis in intramuscular fat of these animals.

*DMD* encodes for dystrophin protein, which has a relevant role in structural function stabilizing the sarcolemma and anchoring the extracellular matrix to the cytoskeleton via F-actin [66]. The authors of [67] suggested that a decrease in the activity of this essential protein may result in progressive porcine *Biceps femoris* muscle degeneration and wasting. Network #6 (Figure 2) shows that *DMD* could activate *MYH8*. Therefore, higher expression of *DMD* seems to result in a better assembly of actin filament binding, which could be more resistant to degradation by proteases hindering the meat tenderization.

*Fos proto-oncogene* (*FOS*) belongs to the immediate early gene family of transcription factors. *FOS* is involved in the maintenance of cytoskeleton, cell-grown regulation, proliferation and differentiation [68]. *FOS* gene maps in a QTL for skeletal muscle fiber detected in a Meishan x Pietrain F2 [69] and codifies for a transcription factor involved that has been previously identified as regulating myogenesis [70]. Differential expression of this gene on muscle has been observed between different breeds divergent for growth and meat quality at different age stages [13,71,72]. In the current study, functional analyses related this gene with skeletal muscle tissue development and cell differentiation (Table 2) as well as connective tissue development (Figure 1). Moreover, IPA analysis showed that *FOS* participates on the IGF-1 signaling pathway (Table S7), which is involved in the activation of receptor tyrosine kinase activity, thereby initiating cell proliferation, cell differentiation and cell survival [73,74] also is an important regulator of cellular growth and metabolism [73].

### 4.3. Lipid Metabolism

It is well known that the intramuscular fat (IMF) content is a main determinant of tenderness in pig. The positive relation between IMF and tenderness could be due to fat cell expansion that may open the muscle structure favoring the muscle separation [75]. However, this relationship is controversial, and it is very influenced by the pig breed [52]. Both IMF and tenderness are heritable traits and the positive genetic correlation among them suggests a common genetic background between IMF and tenderness has also been reported by several authors [76,77]. In addition, some transcriptome studies have shown that genes encoding proteins implicated in IMF accretion are overexpressed in tender pork [78] and it was also proposed that higher IMF content could ease the tenderization associated with the cooking process [78].

Interestingly, our results showed some DEGs involved in biological process related with lipid metabolism that are overexpressed in animals with tenderer meat such as *Fatty Acid Binding Protein 3 (FABP3)* and *Peroxisome Proliferator-Activated Receptor Gamma Coactivator 1-Alpha (PPARGC1A)*. *FABP3* encodes for a member of the fatty acid-binding protein family that comprises a group of small cytosolic proteins, which specifically bind and transport intracellular fatty acids. There are several studies that find associations between polymorphisms in *FABP3* gene and IMF in different pig breeds [79,80,81]. Besides, [80] reported associations between polymorphisms mapped in the *FABP3* gene and tenderness, and a positive correlation between the expression of this gene and IMF in muscle of a Korean x Yorkshire F2. *PPARGC1A* codifies for a transcription factor which regulates hormone receptors and transcription factors involved in adipogenesis and adipocyte differentiation [82] also promotes the fiber conversion to oxidative-type ones [83]. Therefore, this protein could be related with tenderness not only favoring the adipogenesis and IMF content but also for its influence in muscle fiber composition. Actually, there are several studies that report association between polymorphisms located in this gene and tenderness in a commercial hybrid pig population [84]. In the current study, Figure 1 shows as *PPARGC1A* activates *FABP3* and *CTSC* that could suggest favoring the adipogenesis and proteolysis in the group with tenderer meat. Furthermore, *PPARGC1A* is involved in PPARα/RXRα Activation and White Adipose Tissue Browning pathways, which presented a trend for activation in the Tender group and are related with lipid metabolism. Peroxisome proliferator-activated receptor-α (PPARα) heterodimerizes with retinoid x receptor (RXR) and play a role in the transcription of regulator genes of adipocyte differentiation and fatty acid oxidation [85].

It is well known that there is a moderate antagonism between muscular development and intramuscular fat content (IMF) in pigs [86]. In the previous study carried out in the same pig population [24], an overexpression of genes related with myogenesis and skeletal muscle development on animals with low IMF content such as *ACTC1*, *DMD* and *FOS* was observed. As we previously mentioned, our findings indicate that *ACTC1*, *DMD* and *FOS* are upregulated in the Tough group, supporting the hypothesis that IMF is related to the tenderization process in Iberian pigs.

### 4.4. Collagen Metabolic Process

Collagen protein determines the structural support and strength of the extracellular matrix in the connective tissue [87]. Collagen content depends on animal species and age. For instance, collagen crosslinks in older animals is considered related with tougher meat [88] and meat tenderness usually decreases when animals are older as well. Therefore, collagen content seems to contribute to meat toughness. High correlation between collagen content and shear force values measured with Warner–Bratzler method on cattle were found [89]. However, other studies have observed lower correlations in different cattle breeds and ages [90,91]. In a theoretical study, [92] revealed that meat can be ranked in terms of tenderness using the number of collagen crosslink per volume of cooked meat.

In our study, we detected higher expression of several collagen-encoding genes in tougher meat samples compared with tenderer samples, suggesting a differentiation in collagen constituents between divergent samples for shear force. Our gene ontology analysis revealed that cluster 3 (Figure S2) contained DEGs from the collagen family (*COL1A1*, *COL1A2*, *COL12A1* and *COL14A1*), upregulated in the Tough group. Among these DEGs cited previously, *Collagen Type XIV Alpha 1 Chain* (*COL14A1)* encodes for a protein that plays a key role in the extracellular matrix structure organization, cell-cell adhesion and collagen fibril organization [93]. Other authors have also reported differential expression of *COL14A1* between pigs that, a priori, can be divergent for meat tenderness. In the same sense, here, [57] showed that *COL14A1* was upregulated in the transcriptome of Duroc x Iberian pigs compared with Iberian purebred pigs, which are expected to have tenderer meat. In addition, higher expression of this gene was observed in Yorkshire pigs than in Wannanhua [58] and Wei [94] pig breeds with better meat quality properties.

In summary, the use of two different bioinformatics software for functional analysis showed that some of the most significant differential expressed genes encode proteins that have been involved in similar relevant biological functions, networks and pathways. Genes encoding for proteins involved in proteolysis and activators of the conversion of muscle to meat in *post-mortem* process are overexpressed in tenderer meat. Otherwise, those genes codifying for proteins that activate myogenesis, stimulate the muscle development and constitute the extracellular matrix of connective tissue are overexpressed in tougher meat.

The results here are very relevant and support that part of the tenderness variability can be explained by genetics. However, tenderness is a complex trait that can be affected by pre-slaughter conditions as stress situations and other post-mortem factors as temperature [1]. These factors should also be always controlled to avoid undesirable meat textures.

### 4.5. Transcription Regulatory Factors

A study of the potential regulatory factors explaining the observed expression differences between groups was also carried out. It is not necessary that the regulatory factors are differentially expressed since they can join to DNA sequences adjacent to DEGs with more or less affinity due to potential mutations located in these DNA motifs or in coding sequences of the regulatory factors that could alter the final protein structure.

The IPA analyses predicted a regulator effect network that could explain the expression of some DEGs. Figure 3 represented causal hypotheses to interpret the regulatory potential mechanism of the upstream regulator IGF1 on *FOS, FN1, COL1A1* and *THY1*. Apparently, IGF1 activates the expression of *FOS, FN1, COL1A1* and *THY1* that are overexpressed in Iberian pigs with tougher meats and inhibits the expression of CCAAT/enhancer binding protein delta (*CEBPD*) and proteasome subunit beta 8 (*PSMB8*) which are repressed in this type of pigs. As was explained above, *FOS* is involved in muscle growth and development and *COL1A1* in the extracellular matrix constitution; therefore, a higher activation of this process seems to make tougher meat. On the other hand, *CEBPD* plays an essential role during the earliest phases of the adipocyte differentiation [95] and *PSMB8* maps in a genomic region explaining part of the IMF phenotypical variance observed in Iberian pigs [96]. Therefore, IGF1 would activate the muscle growth and inhibit adipogenesis explaining the antagonism relationship between these traits.

One of the most significant regulatory factors is vestigial-like family member 3 (VGLL3) (Table 5), which was identified as a transcriptional co-factor associated with myogenesis, skeletal muscle development and muscle hypertrophy [97]. VGLL3 was predicted to be activated in tougher samples inducing the expression of *COL12A1, COL1A1*, *COL1A2* and *GADD45B* genes. Some of them are involved in collagen metabolic process (*COL12A1, COL1A1*, and *COL1A2*), cellular growth and apoptosis (*GADD45B*). Therefore, a higher activity of this transcription factor would hinder the tenderization process.

Peroxisome proliferator-activated receptor gamma (PPARG) is a ligand-dependent nuclear receptor known as the “master regulator of adipogenesis”, being related with lipid metabolism processes as adipose differentiation [98] and it has been identified as a potential candidate genes for improving IMF content [99]. Moreover, a higher expression of PPARG gene have been observed in Iberian piglets [100] and foetuses [72] than in Duroc x Iberian piglets and Large-White foetuses, respectively, which have less IMF content than Iberian ones. In this study, although the expression differences of this gene were not observed, the upstream analysis identified this gene as an activator of *FABP3* and *PPARGC1A*, which, as has been pointed out above, are overexpressed in the Tender group and promote adipogenesis and increase IMF content.

### 4.6. Candidate Genes for an Iberian Pig-Breeding Program

The ultimate objective of this study was to propose several candidate genes for searching polymorphisms and design a genotyping panel for improving tenderness in Iberian pigs. In further steps, polymorphisms with divergent allelic frequencies should be identified in the regulatory regions of the proposed candidate genes and association analyses between their genotypes and shear force should be carried out in the same Iberian pig population.

In summary, the most promising candidate genes to be selected are involved in proteolysis processes (*ACTC1, ANKRD1, CHRNA9, CTSC* and *RHOD*), skeletal muscle tissue development and growth (*DMD* and *FOS*), lipid metabolism (*FABP3* and *PPARGC1A*) and collagen metabolic process (*COL14A1*). Although the *MSTN* gene is clearly involved in muscle growth, the results observed here are controversial since this protein inhibits myogenesis and a higher expression in the Tough group was observed; therefore, more cautions should be taken before to be included as a selection marker.

It is also interesting to consider genes encoding regulator factors such as *IGF1*, *PPARG* and *VGLL3* since they modulate the expression of some of the genes mentioned before.

It is worth mentioning that some genes, such as *ACTC1, DMD* and *FOS*, were also overexpressed in Iberian pigs with low IMF content (Muñoz et al., 2018); therefore, they could be used for improving both IMF content as shear force (tenderness).

## 5. Conclusions

In our study, we identified 200 differentially expressed annotated genes and 245 newly predicted isoforms on the LD muscle transcriptome of 13 Iberian pigs with divergent breeding values for tenderness measured through data of shear force with Warner–Bratzler analysis. The use of two different pieces of bioinformatics software for the functional analysis of these DEGs has revealed relevant biological processes, canonical pathways and networks potentially related with tenderness. The most representative functions associated with this trait are proteolysis, skeletal muscle development, lipid metabolism and collagen metabolism. Generally, genes encoding for proteins involved in proteolysis and conversion of muscle to meat (*ANKRD1*, *CASQ2*, *CHRNA9*, *CTSC*, and *RHOD*) are overexpressed in the Tender group while genes encoding for proteins enhancing myogenesis and muscle development (*FOS* and *DMD*) are overexpressed in the Tough one. In addition to this, genes involved in lipid (*FABP3* and *PPARGC1A*) and collagen metabolisms (*COL14A1*) are also relevant. Additionally, the upstream analysis has identified several transcriptional regulatory factors (IGF1, PPARG and VGLL3) that regulate the expression of some differentially expressed genes mentioned before, such as *FOS* or *COL1A1.*

This study is a first approach to understand the biological mechanisms underlying the trait meat tenderness and it provides a set of candidate genes that could harbor polymorphisms affecting tenderness in Iberian pigs. However, further studies including functional analyses such as immunohistochemical staining and/or Western blot analysis should be performed to experimentally validate if the proteins codified by the proposed candidate genes are responsible of the variation in tenderness. Additional steps, such as identifying polymorphisms with opposed allelic frequencies in the extreme groups, performing association analyses between the identified polymorphisms and tenderness and assessing their effects on other quality meat and productive traits, should be carried out to apply this information in a breeding program to improve tenderness in the Iberian pig.

## Figures and Tables

**Table 1:** Fold change, mean expression value in the Tender and Tough groups, *q-value* and with *p-value < 0.05*, corresponding to the most relevant differentially expressed genes.

Gene	Fold Change	Tender	Tough	*q*-Value
*Guanylate binding protein 1 (GBP1)*	0.089	46.959	4.175	0.002
*Cholinergic Receptor Nicotinic Alpha 9 Subunit (CHRNA9)*	0.360	1.666	0.600	0.002
*Ras Homolog Family Member D (RHOD)*	0.438	1.414	0.620	0.025
*Calsequestrin 2 (CASQ2)*	0.447	15.284	6.832	0.002
*Ankyrin Repeat Domain 1 protein (ANKRD1)*	0.476	553.625	263.267	0.002
*Peroxisome Proliferator-Activated Receptor Gamma Coactivator 1-Alpha (PPARGC1A)*	0.508	13.825	7.024	0.015
*Cathepsine C (CTSC)*	0.567	63.679	36.137	0.016
*Fatty Acid Binding Protein 3 (FABP3)*	0.633	352.059	222.898	0.038
*Fos proto-oncogene (FOS)*	1.670	24.722	41.282	0.037
*Dystrophin (DMD)*	1.779	0.614	1.091	0.038
*Collagen Type XIV Alpha 1 Chain (COL14A1)*	2.017	3.035	6.122	0.057
*Myostatin (MSTN)*	2.038	2.977	6.067	0.009
*Growth Arrest and DNA Damage Inducible Beta protein (GADD45B)*	2.871	12.954	37.188	0.002
*Actin Alpha 1, Skeletal Muscle (ACTC1)*	4.085	9.750	39.830	0.002
*Myosin Heavy Chain 8 (MYH8)*	7.541	1269.750	9574.930	0.002
*Family With Sequence Similarity 180 Member B (FAM180B)*	8.830	1.959	17.297	0.008

Mean expression values are expressed in fragments per kilobase of transcript per million mapped fragments (FPKMs).

**Table 2:** Summary of the most relevant significantly overrepresented Gene Ontology (GO) terms related with tenderness on differentially expressed genes (DEGs) using FatiGO.

Term	Genes	Adjusted *p*-Value
GO_BP_		
Skeletal muscle tissue development (GO:0007519)	*MYLK2, MSTN, FOS, HLF, CXCL10, IGFBP5, ANKRD1, DMD, CXCL9, FOXN2*	1.02 × 10^−9^
Muscle cell development (GO:0055001)	*CXCL10, ANKRD1, DMD, CXCL9, CASQ2, ACTC1, COL14A1*	9.94 × 10^−6^
Skeletal muscle cell differentiation (GO:0035914)	*MYLK2, FOS, HLF, ANKRD1, FOXN2*	9.94 × 10^−6^
Regulation of muscle system process (GO:0090257)	*MYLK2, MSTN, CTGF, DMD, ADRA2C, CASQ2, COL14A1*	3.72 × 10^−5^
Collagen metabolic process (GO:0032963)	*CTGF, COL1A2, COL1A1, ENG, COL12A1, COL14A1*	3.72 × 10^−5^
Response to amino acid (GO:0043200)	*CTGF, COL1A2, COL1A1, CDO1, PPARGC1A*	4.07 × 10^−5^
Cytosolic calcium ion transport (GO:0060401)	*CTGF, CXCL10, DMD, CXCL9, THY1, CASQ2*	6.75 × 10^−5^
Regulation of muscle tissue development (GO:1901861)	*MSTN, PPARGC1A, CXCL10, CXCL9, COL14A1*	3.70 × 10^−4^
Regulation of calcium ion transport (GO:0051924)	*CXCL10, DMD, CXCL9, ATP2B2, THY1, CASQ2*	4.40 × 10^−4^
Actin-myosin filament sliding (GO:0033275)	*MYLK2, MYH8, DMD, ACTC1*	4.53 × 10^−4^
JNK cascade (GO:0007254)	*CTGF, SFRP4, PAK1, TRIB1, DUSP10, GADD45B*	4.53 × 10^−4^
Negative regulation of protein kinase activity (GO:0006469)	*PPP1R1B, THY1, DUSP1, TRIB1, DUSP10, GADD45B*	5.19 × 10^−4^
Collagen fibril organization (GO:0030199)	*COL1A2, COL1A1, COL12A1, COL14A1*	1.33 × 10^−4^
Actin-mediated cell contraction (GO:0070252)	*MYLK2, MYH8, DMD, ACTC1*	1.11 × 10^−3^
Regulation of muscle contraction (GO:0006937)	*MYLK2, CTGF, DMD, ADRA2C, CASQ2*	1.24 × 10^−3^
Regulation of JNK cascade (GO:0046328)	*CTGF, SFRP4, PAK1, DUSP10, GADD45B*	1.27 × 10^−3^
Regulation of stress-activated MAPK cascade (GO:0032872)	*CTGF, SFRP4, PAK1, DUSP10, GADD45B*	2.26 × 10^−3^
Regulation of stress-activated protein kinase signaling cascade (GO:0070302)	*CTGF, SFRP4, PAK1, DUSP10, GADD45B*	2.27 × 10^−3^
Skeletal muscle tissue growth (GO:0048630)	*MSTN, IGFBP5*	2.97 × 10^−3^
Regulation of protein kinase B signaling (GO:0051896)	*SLC9A3R1, ITSN1, IGFBP5, RASD2*	3.48 × 10^−3^
Actin filament bundle organization (GO:0061572)	*RHOD, CTGF, PAK1, PFN2*	4.82 × 10^−3^
Positive regulation of proteolysis involved in cellular protein catabolic process (GO:1903052)	*ZFAND2A, CTSC, TRIB1*	7.85 × 10^−3^
Actomyosin structure organization (GO:0031032)	*ANKRD1, CASQ2, ACTC1*	1.61 × 10^−2^

**Table 3:** List of relevant enriched networks and functions related with tenderness identified in the set of DEGs between the Tender and Tough groups identified by IPA software. Genes showing the highest expression differences between groups are in bold.

ID	Molecules in Network	Score	Focus Molecules	Functions
5	**ADCY1**, AMPK, **ANGPTL4**, Ap1, Cdk, CG, **CPLX1**, Creb, **CRHR2**, **CTSC**, **CXCL10**, cytochrome C, cytokine, **DUSP1**, **FABP3**, **FOS**, Insulin, **KRT80**, **LPL**, **MAF**, Mapk, Mek, NRN1, P38 MAPK, PARP, PDGF BB, Pkc(s), **PPARGC1A**, PRKAA, **RUNX1**, **SLC25A33**, **SOX6**, TCR, Vegf, VLDL-cholesterol	26	17	Connective Tissue Development and Function, Lipid Metabolism, Tissue Morphology
6	**ACTC1**, **AHSP**, **AQP4**, **ARPP21**, BAZ1A, **CCL4**, CD3, CHRNA10, **CHRNA9**, **CRABP2**, **DMD**, ERK, FLNC, FSH, GK, Histone h3, Histone h4, **HLF**, IgG, Jnk, Lh, miR-130a-3p (and other miRNAs w/seed AGUGCAA), mir-672, **MYH8**, **MYLK2**, Nr1h, RNA polymerase II, **SLC9A7**, Smad2/3, Sos, **THY1**, TMEM184A, **TRIB1**, **WFDC1**, **ZIC1**	23	16	Cell Morphology, Cellular Assembly and Organization, Cellular Function and Maintenance

**Table 4:** List of significant pathways (*p*-value < 0.05) with assigned z-score identified in the set of DEGs according to the Tender and Tough group identified by Ingenuity Pathway Analysis (IPA) software.

Canonical Pathways	*p*-Value	Ratio	z-Score	Molecules
Integrin Signaling	0.001	0.033	0.447	ACTC1, MYLK2, PAK1, PFN2, RASD2, RHOBTB1, RHOD
Hepatic Fibrosis Signaling Pathway	0.001	0.027	1.265	CCN2, COL1A1, COL1A2, FOS, MYLK2, RASD2, RHOBTB1, RHOD, TFRC, YAP1
Actin Cytoskeleton Signaling	0.002	0.032	1.342	ACTC1, FN1, MYH8, MYLK2, PAK1, PFN2, RASD2
Synaptogenesis Signaling Pathway	0.003	0.026	−0.378	ADCY1, ADCY6, CPLX1, ITSN1, MARCKS, PAK1, RASD2, SNCG
PPARα/RXRα Activation	0.004	0.032	−1.342	ADCY1, ADCY6, GPD2, LPL, PPARGC1A, RASD2
ILK Signaling	0.004	0.032	1.342	ACTC1, FN1, FOS, MYH8, RHOBTB1, RHOD
Tec Kinase Signaling	0.009	0.031	1	ACTC1, FOS, PAK1, RHOBTB1, RHOD
Cardiac Hypertrophy Signaling	0.011	0.025	−1	ADCY1, ADCY6, ADRA2C, RASD2, RHOBTB1, RHOD
GNRH Signaling	0.011	0.029	1	ADCY1, ADCY6, FOS, PAK1, RASD2
Signaling by Rho Family GTPases	0.012	0.025	0.447	ACTC1, CIT, FOS, PAK1, RHOBTB1, RHOD
RhoA Signaling	0.015	0.033	−1	ACTC1, CIT, MYLK2, PFN2
White Adipose Tissue Browning Pathway	0.018	0.031	−1	ADCY1, ADCY6, LDHB, PPARGC1A

Ratio: number of DEGs in a pathway divided by the number of genes comprised in the same pathway.

**Table 5:** List of significant upstream regulators identified in the set of DEGs according to the Tender and Tough group (*p*-value < 0.05 and z-score > 2 or < −2).

Upstream Regulator	Molecule Type	PAS	Activation z-Score	*p*-Value of Overlap	Molecules in Dataset	Related Functions
IGF1	Growth factor	Activated	2.947	7.29 × 10^−8^	CCL4, CEBPD, COL1A1, DUSP1, FN1, FOS, IGFBP5, LPL, MYH8, PSMB8	Development of body axis
VGLL3	Other	Activated	2.000	1.99 × 10^−6^	COL12A1, COL1A1, COL1A2, GADD45B	
SEMA7A	Transmembrane receptor	Activated	2.000	3.31 × 10^−5^	CCN2, COL1A1, COL1A2, FN1	
PTH	Other	Activated	2.197	4.51 × 10^−5^	COL1A1, COL1A2, DUSP1, FOS, IGFBP5, SFRP4	
KLF11	Transcription regulator	Inhibited	−2.236	4.92 × 10^−4^	CCN2, COL1A2, CPT2, ENG, FABP3, PPARGC1A	
IL4	Cytokine	Inhibited	−2.331	5.93 × 10^−4^	ALDOC, CCL26, CCL4, CD163, CXCL10, FOS, LPL, NABP1, PPARGC1A, TFRC	
TRIM24	Transcription regulator	Activated	2.236	1.30 × 10^−3^	CXCL10, PSMB10, PSMB8, PSMB9, TAP1	
PPARG	Ligand-dependent nuclear receptor	Inhibited	−2.179	1.89 × 10^−3^	ANGPTL4, COL1A1, COL1A2, CPT2, CRABP2, FABP3, FN1, IGFBP5, LPL, PPARGC1A	
OGT	Enzyme	Inhibited	−2.000	2.42 × 10^−3^	FOS, LPL, PPARGC1A, THY1	
NOS2	Enzyme	Inhibited	−2.219	3.13 × 10^−3^	ACTC1, CCL4, CTSC, CYCS, PPARGC1A, THY1	
SATB1	Transcription regulator	Activated	2.000	4.18 × 10^−2^	GADD45B, HBB, MAF, RUNX1	

PAS: Predicted Activation State, predicted Activated in the Tough group (z-Score > 2), predicted Inhibited in the Tough group (z-Score < −2).

**Table 6:** Technical validation of RNA-seq results by quantitative PCR (qPCR): Fold Change values (FC), Pearson correlations (r^2^) and Concordance Correlation Coefficient (CCC) between expression values obtained from both techniques.

Gene	Expression Type	qPCR FC	RNAseq FC	r^2^	*p*-Value	CCC
*ACTC1*	Tender < Tough	2.861	4.085	0.986	6.20 × 10^−10^	0.828
*MX1*	Tender < Tough	2.231	2.195	0.943	1.34 × 10^−6^	
*COL1A1*	Tender < Tough	1.503	1.636	0.922	7.29 × 10^−6^	
*FOS*	Tender < Tough	1.634	1.670	0.913	1.29 × 10^−5^	
*ANKRD1*	Tender > Tough	0.531	0.476	0.779	0.002	
*MSTN*	Tender < Tough	1.753	2.038	0.527	0.064	
*IRF1*	NO DE	0.656	0.235	0.997	1.81 × 10^−13^	
*NOS2*	NO DE	0.611	0.811	0.701	0.008	
*SSH2*	NO DE	1.037	1.625	0.584	0.036	
*ELOVL6*	NO DE	0.887	1.712	0.565	0.044	

NO DE: No differentially expressed in RNA-seq experiment. Tender > Tough: higher expression in the Tender than in the Tough group. Tender < Tough: lower expression in the Tender than in the Tough group.

## Supplementary Materials

The following are available online at www.mdpi.com/xxx/s1, Table S1: Primer name, sequences, melting temperature (TM) and amplicon size of genes selected for qPCR validation, Table S2: Total number of reads, filtered reads, and percentage of mapped reads per sample, Table S3: Classification of the transcripts identified in the *Longissimus dorsi* muscle of Iberian pigs in relation to the Ensembl annotated pig genes, Table S4: Fold change, mean expression value in the Tender and Tough groups, *p*-value, and *q*-value corresponding to DEGs and differentially expressed novel isoforms, Table S5: List of overrepresented GO terms on DEGs between the Tender and Tough groups using FatiGO, Table S6: Complete list of networks, diseases and functions identified on DEGs between the Tender and Tough groups by IPA software, Table S7: Ingenuity Pathway Analysis (IPA). List of significant pathways (*p*-value < 0.05) identified in the set of DEGs according to the Tender and Tough groups, Table S8: Ingenuity Pathway Analysis (IPA). List of upstream regulators identified in the set of DEGs according to the Tender and Tough group (*p*-value < 0.05). PAS: Predicted activation ratio. Figure S1: Gene expression distribution of the 25,878 genes annotated in the pig genome (Sscrofa11.1) in fragments per kilobase of transcript per million mapped fragments (FPKMs) normalized values corresponding to the animals with the highest EBV for shear force and tougher meat (High EBV SFF) and the lowest EBV for shear force and tenderer meat (Low EBV SFF). Figure S2: Network of protein–protein interactions predicted with the STRING database. Same color nodes sharing multiple edges are grouped in the same cluster. Figure S3: Gene network #8: Cardiovascular System Development and Function, Cell Cycle, Gene Expression. Genes upregulated and down-regulated in the Tender group are represented in green and red colors, respectively. Figure S4: Gene network #9: Cell Cycle, Cellular Assembly and Organization, Cellular Movement. Genes upregulated and down-regulated in the Tender group are represented in green and red colors, respectively.
