# Supplementary material for: Differences in the Loin Tenderness of Iberian Pigs Explained through Dissimilarities in Their Transcriptome Expression Profile
Source: Animals (Basel). 2020 Sep 22;10(9):0. doi: 10.3390/ani10091715 (PMC7552750; doi:10.3390/ani10091715)
Supplement: Supplementary file 1 [file animals-10-01715-s001.zip › Tables_and_SupTables/1 TABLES - format.docx]

**Table 1**. Fold change, mean expression value on Tender and Tough groups, *p-value*, and *q-value* corresponding to the most relevant differentially expressed genes.

| Gene | Fold change | Tender | Tough | *p-value* | *q-value* |
| --- | --- | --- | --- | --- | --- |
| *Guanylate binding protein 1 (GBP1)* | 0.089 | 46.959 | 4.175 | 5.00x10^-05^ | 0.002 |
| *Cholinergic Receptor Nicotinic Alpha 9 Subunit (CHRNA9)* | 0.360 | 1.666 | 0.600 | 5.00x10^-05^ | 0.002 |
| *Ras Homolog Family Member D (RHOD)* | 0.438 | 1.414 | 0.620 | 1.95x10^-03^ | 0.025 |
| *Calsequestrin 2 (CASQ2)* | 0.447 | 15.284 | 6.832 | 5.00x10^-05^ | 0.002 |
| *Ankyrin Repeat Domain 1 protein (ANKRD1)* | 0.476 | 553.625 | 263.267 | 5.00x10^-05^ | 0.002 |
| *Peroxisome Proliferator-Activated Receptor Gamma Coactivator 1-Alpha (PPARGC1A)* | 0.508 | 13.825 | 7.024 | 9.00x10^-04^ | 0.015 |
| *Cathepsine C (CTSC)* | 0.567 | 63.679 | 36.137 | 9.50x10^-04^ | 0.016 |
| *Fatty Acid Binding Protein 3 (FABP3)* | 0.633 | 352.059 | 222.898 | 3.65x10^-03^ | 0.038 |
| *Fos proto-oncogene (FOS)* | 1.670 | 24.722 | 41.282 | 3.25x10^-03^ | 0.037 |
| *Dystrophin (DMD)* | 1.779 | 0.614 | 1.091 | 3.40x10^-03^ | 0.038 |
| *Collagen Type XIV Alpha 1 Chain (COL14A1)* | 2.017 | 3.035 | 6.122 | 6.55x10^-03^ | 0.057 |
| *Myostatin (MSTN)* | 2.038 | 2.977 | 6.067 | 4.50x10^-04^ | 0.009 |
| *Growth Arrest and DNA Damage Inducible Beta protein (GADD45B)* | 2.871 | 12.954 | 37.188 | 5.00x10^-05^ | 0.002 |
| *Actin Alpha 1, Skeletal Muscle (ACTC1)* | 4.085 | 9.750 | 39.830 | 5.00x10^-05^ | 0.002 |
| *Myosin Heavy Chain 8 (MYH8)* | 7.541 | 1,269.750 | 9,574.930 | 5.00x10^-05^ | 0.002 |
| *Family With Sequence Similarity 180 Member B (FAM180B)* | 8.830 | 1.959 | 17.297 | 3.50x10^-04^ | 0.008 |

Mean expression values are expressed in fragments per kilobase of transcript per million mapped fragments (FPKMs)*.*

**Table 2.** Summary of the most relevant significantly overrepresented GO terms related with tenderness on DEGs using FatiGO.

| Term | Genes | Adjusted *p-value* |
| --- | --- | --- |
| GO_BP_ |  |  |
| Skeletal muscle tissue development(GO:0007519) | *MYLK2, MSTN, FOS, HLF, CXCL10, IGFBP5, ANKRD1, DMD, CXCL9, FOXN2* | 1.02x10^-09^ |
| Muscle cell development(GO:0055001) | *CXCL10, ANKRD1, DMD, CXCL9, CASQ2, ACTC1, COL14A1* | 9.94x10^-06^ |
| Skeletal muscle cell differentiation(GO:0035914) | *MYLK2, FOS, HLF, ANKRD1, FOXN2* | 9.94x10^-06^ |
| Regulation of muscle system process(GO:0090257) | *MYLK2, MSTN, CTGF, DMD, ADRA2C, CASQ2, COL14A1* | 3.72x10^-05^ |
| Collagen metabolic process(GO:0032963) | *CTGF, COL1A2, COL1A1, ENG, COL12A1, COL14A1* | 3.72x10^-05^ |
| Response to amino acid(GO:0043200) | *CTGF, COL1A2, COL1A1, CDO1, PPARGC1A* | 4.07x10^-05^ |
| Cytosolic calcium ion transport(GO:0060401) | *CTGF, CXCL10, DMD, CXCL9, THY1, CASQ2* | 6.75x10^-05^ |
| Regulation of muscle tissue development(GO:1901861) | *MSTN, PPARGC1A, CXCL10, CXCL9, COL14A1* | 3.70x10^-04^ |
| Regulation of calcium ion transport(GO:0051924) | *CXCL10, DMD, CXCL9, ATP2B2, THY1, CASQ2* | 4.40x10^-04^ |
| Actin-myosin filament sliding(GO:0033275) | *MYLK2, MYH8, DMD, ACTC1* | 4.53x10^-04^ |
| JNK cascade(GO:0007254) | *CTGF, SFRP4, PAK1, TRIB1, DUSP10, GADD45B* | 4.53x10^-04^ |
| Negative regulation of protein kinase activity(GO:0006469) | *PPP1R1B, THY1, DUSP1, TRIB1, DUSP10, GADD45B* | 5.19x10^-04^ |
| Collagen fibril organization(GO:0030199) | *COL1A2, COL1A1, COL12A1, COL14A1* | 1.33x10^-04^ |
| Actin-mediated cell contraction(GO:0070252) | *MYLK2, MYH8, DMD, ACTC1* | 1.11x10^-03^ |
| Regulation of muscle contraction(GO:0006937) | *MYLK2, CTGF, DMD, ADRA2C, CASQ2* | 1.24x10^-03^ |
| Regulation of JNK cascade(GO:0046328) | *CTGF, SFRP4, PAK1, DUSP10, GADD45B* | 1.27x10^-03^ |
| Regulation of stress-activated MAPK cascade(GO:0032872) | *CTGF, SFRP4, PAK1, DUSP10, GADD45B* | 2.26x10^-03^ |
| Regulation of stress-activated protein kinase signaling cascade(GO:0070302) | *CTGF, SFRP4, PAK1, DUSP10, GADD45B* | 2.27x10^-03^ |
| Skeletal muscle tissue growth(GO:0048630) | *MSTN, IGFBP5* | 2.97x10^-03^ |
| Regulation of protein kinase B signaling(GO:0051896) | *SLC9A3R1, ITSN1, IGFBP5, RASD2* | 3.48x10^-03^ |
| Actin filament bundle organization(GO:0061572) | *RHOD, CTGF, PAK1, PFN2* | 4.82x10^-03^ |
| Positive regulation of proteolysis involved in cellular protein catabolic process(GO:1903052) | *ZFAND2A, CTSC, TRIB1* | 7.85x10^-03^ |
| Actomyosin structure organization(GO:0031032) | *ANKRD1, CASQ2, ACTC1* | 1.61x10^-02^ |

**Table 3**. List of relevant enriched networks and functions related with tenderness identified in the set of DEGs between Tender and Tough groups identified by IPA software

| ID | Molecules in network | Score | Focus Molecules | Functions |
| --- | --- | --- | --- | --- |
| 5 | **ADCY1**, AMPK, **ANGPTL4**, Ap1, Cdk, CG, **CPLX1**, Creb, **CRHR2**, **CTSC**, **CXCL10**, cytochrome C, cytokine, **DUSP1**, **FABP3**, **FOS**, Insulin, **KRT80, LPL**, **MAF**, Mapk, Mek, **NRN1**, P38 MAPK, PARP, PDGF BB, Pkc(s), **PPARGC1A**, PRKAA, **RUNX1**, **SLC25A33**, **SOX6**, TCR, Vegf, VLDL-cholesterol | 26 | 17 | Connective Tissue Development and Function, Lipid Metabolism, Tissue Morphology |
| 6 | **ACTC1**, **AHSP**, **AQP4**, **ARPP21**, BAZ1A, **CCL4**, CD3, CHRNA10, **CHRNA9**, **CRABP2**, **DMD**, ERK, FLNC, FSH, GK, Histone h3, Histone h4, **HLF**, IgG, Jnk, Lh, miR-130a-3p (and other miRNAs w/seed AGUGCAA), mir-672, **MYH8**, **MYLK2**, Nr1h, RNA polymerase II, **SLC9A7**, Smad2/3, Sos, **THY1**, TMEM184A, **TRIB1**, **WFDC1**, **ZIC1** | 23 | 16 | Cell Morphology, Cellular Assembly and Organization, Cellular Function and Maintenance |

Genes showing the highest expression differences between groups are in bold.

**Table 4**. List of significant pathways (*p*-value < 0.05) with assigned z-score identified in the set of DEGs according to Tender and Tough group identified by IPA software

| **Canonical Pathways** | ***p-value*** | **Ratio** | **z-Score** | **Molecules** |
| --- | --- | --- | --- | --- |
| Integrin Signaling | 0.001 | 0.033 | 0.447 | ACTC1, MYLK2, PAK1, PFN2, RASD2, RHOBTB1, RHOD |
| Hepatic Fibrosis Signaling Pathway | 0.001 | 0.027 | 1.265 | CCN2, COL1A1, COL1A2, FOS, MYLK2, RASD2, RHOBTB1, RHOD, TFRC, YAP1 |
| Actin Cytoskeleton Signaling | 0.002 | 0.032 | 1.342 | ACTC1, FN1, MYH8, MYLK2, PAK1, PFN2, RASD2 |
| Synaptogenesis Signaling Pathway | 0.003 | 0.026 | -0.378 | ADCY1, ADCY6, CPLX1, ITSN1, MARCKS, PAK1, RASD2, SNCG |
| PPARα/RXRα Activation | 0.004 | 0.032 | -1.342 | ADCY1, ADCY6, GPD2, LPL, PPARGC1A, RASD2 |
| ILK Signaling | 0.004 | 0.032 | 1.342 | ACTC1, FN1, FOS, MYH8, RHOBTB1, RHOD |
| Tec Kinase Signaling | 0.009 | 0.031 | 1 | ACTC1, FOS, PAK1, RHOBTB1, RHOD |
| Cardiac Hypertrophy Signaling | 0.011 | 0.025 | -1 | ADCY1, ADCY6, ADRA2C, RASD2, RHOBTB1, RHOD |
| GNRH Signaling | 0.011 | 0.029 | 1 | ADCY1, ADCY6, FOS, PAK1, RASD2 |
| Signaling by Rho Family GTPases | 0.012 | 0.025 | 0.447 | ACTC1, CIT, FOS, PAK1, RHOBTB1, RHOD |
| RhoA Signaling | 0.015 | 0.033 | -1 | ACTC1, CIT, MYLK2, PFN2 |
| White Adipose Tissue Browning Pathway | 0.018 | 0.031 | -1 | ADCY1, ADCY6, LDHB, PPARGC1A |

Ratio: number of DEGs in a pathway divided by the number of genes comprised in the same pathway.

**Table 5.** List of significant upstream regulators identified in the set of DEGs according to Tender and Tough group (p-value < 0.05 and z-score > 2 or < -2).

| **Upstream Regulator** | **Molecule Type** | **PAS** | **Activation z-Score** | ***p-value* of overlap** | **Molecules in Dataset** | **Related Functions** |
| --- | --- | --- | --- | --- | --- | --- |
| IGF1 | Growth factor | Activated | 2.947 | 7.29x10^-8^ | CCL4, CEBPD, COL1A1, DUSP1, FN1, FOS, IGFBP5, LPL, MYH8, PSMB8 | Development of body axis |
| VGLL3 | Other | Activated | 2.000 | 1.99x10^-6^ | COL12A1, COL1A1, COL1A2, GADD45B |  |
| SEMA7A | Transmembrane receptor | Activated | 2.000 | 3.31x10^-5^ | CCN2, COL1A1, COL1A2, FN1 |  |
| PTH | Other | Activated | 2.197 | 4.51x10^-05^ | COL1A1, COL1A2, DUSP1, FOS, IGFBP5, SFRP4 |  |
| KLF11 | Transcription regulator | Inhibited | -2.236 | 4.92x10^-04^ | CCN2, COL1A2, CPT2, ENG, FABP3, PPARGC1A |  |
| IL4 | Cytokine | Inhibited | -2.331 | 5.93x10^-04^ | ALDOC, CCL26, CCL4, CD163, CXCL10, FOS, LPL, NABP1, PPARGC1A, TFRC |  |
| TRIM24 | Transcription regulator | Activated | 2.236 | 1.30x10^-03^ | CXCL10, PSMB10, PSMB8, PSMB9, TAP1 |  |
| PPARG | Ligand-dependent nuclear receptor | Inhibited | -2.179 | 1.89x10^-03^ | ANGPTL4, COL1A1, COL1A2, CPT2, CRABP2, FABP3, FN1, IGFBP5, LPL, PPARGC1A |  |
| OGT | Enzyme | Inhibited | -2.000 | 2.42x10^-03^ | FOS, LPL, PPARGC1A, THY1 |  |
| NOS2 | Enzyme | Inhibited | -2.219 | 3.13x10^-03^ | ACTC1, CCL4, CTSC, CYCS, PPARGC1A, THY1 |  |
| SATB1 | Transcription regulator | Activated | 2.000 | 4.18x10^-02^ | GADD45B, HBB, MAF, RUNX1 |  |

PAS: Predicted Activation State, predicted Activated in Tough group (z-Score > 2), predicted Inhibited in Tough group (z-Score < -2).

**Table 6.** Technical validation of RNAseq results by quantitative PCR (qPCR): Fold Change values (FC), Pearson correlations (r^2^) and Concordance Correlation Coefficient (CCC) between expression values obtained from both techniques.

| Gene | Expression type | qPCR FC | RNAseq FC | r^2^ | *p-value* | CCC |
| --- | --- | --- | --- | --- | --- | --- |
| *ACTC1* | Tender < Tough | 2.861 | 4.085 | 0.986 | 6.20x10^-10^ | 0.828 |
| *MX1* | Tender < Tough | 2.231 | 2.195 | 0.943 | 1.34x10 ^-6^ |  |
| *COL1A1* | Tender < Tough | 1.503 | 1.636 | 0.922 | 7.29x10^-6^ |  |
| *FOS* | Tender < Tough | 1.634 | 1.670 | 0.913 | 1.29x10^-5^ |  |
| *ANKRD1* | Tender > Tough | 0.531 | 0.476 | 0.779 | 0.002 |  |
| *MSTN* | Tender < Tough | 1.753 | 2.038 | 0.527 | 0.064 |  |
| *IRF1* | NO DE | 0.656 | 0.235 | 0.997 | 1.81x10^-13^ |  |
| *NOS2* | NO DE | 0.611 | 0.811 | 0.701 | 0.008 |  |
| *SSH2* | NO DE | 1.037 | 1.625 | 0.584 | 0.036 |  |
| *ELOVL6* | NO DE | 0.887 | 1.712 | 0.565 | 0.044 |  |

NO DE: No differentially expressed in RNAseq experiment. Tender > Tough: higher expression in Tender than in Tough group. Tender < Tough: lower expression in Tender than in Tough group.
